# Disentangling the mechanisms of mate choice in a captive koala population

**DOI:** 10.7717/peerj.5438

**Published:** 2018-08-21

**Authors:** Parice A. Brandies, Catherine E. Grueber, Jamie A. Ivy, Carolyn J. Hogg, Katherine Belov

**Affiliations:** 1 School of Life and Environmental Sciences, University of Sydney, Sydney, NSW, Australia; 2 San Diego Zoo Global, San Diego, CA, USA

**Keywords:** Mate choice, Captive breeding, Genetic compatibility, Major histocompatibility complex (MHC), Male heterozygosity, Microsatellites

## Abstract

Successful captive breeding programs are crucial to the long-term survival of many threatened species. However, pair incompatibility (breeding failure) limits sustainability of many captive populations. Understanding whether the drivers of this incompatibility are behavioral, genetic, or a combination of both, is crucial to improving breeding programs. We used 28 years of pairing data from the San Diego Zoo koala colony, plus genetic analyses using both major histocompatibility complex (MHC)-linked and non-MHC-linked microsatellite markers, to show that both genetic and non-genetic factors can influence mating success. Male age was reconfirmed to be a contributing factor to the likelihood of a koala pair copulating. This trend could also be related to a pair’s age difference, which was highly correlated with male age in our dataset. Familiarity was reconfirmed to increase the probability of a successful copulation. Our data provided evidence that females select mates based on MHC and genome-wide similarity. Male heterozygosity at MHC class II loci was associated with both pre- and post-copulatory female choice. Genome-wide similarity, and similarity at the MHC class II DAB locus, were also associated with female choice at the post-copulatory level. Finally, certain MHC-linked alleles were associated with either increased or decreased mating success. We predict that utilizing a variety of behavioral and MHC-dependent mate choice mechanisms improves female fitness through increased reproductive success. This study highlights the complexity of mate choice mechanisms in a species, and the importance of ascertaining mate choice mechanisms to improve the success of captive breeding programs.

## Introduction

Captive breeding programs contribute to species conservation and are one of the conservation tools used to prevent extinction (Fa, Funk & O’Connell, 2011). The number of endangered and critically endangered species has been growing every year, largely due to human activities (IUCN, 2016). Currently there are almost 25,000 threatened species on the IUCN red list and the need for effective captive breeding programs is greater than ever before (IUCN, 2016). However, approximately 50% of captive populations are not sustainable: animals are not reliably breeding to replacement, nor retaining the required levels of genetic diversity (a goal typically set at ≥90% of wild source gene diversity) (Lees & Wilcken, 2009).

Many captive breeding programs are managed using a mean kinship strategy that aims to pair individuals that are least related to each other (based on the pedigree) (Ballou et al., 2010). Although this strategy has been widely applied through the zoo industry for the past few decades (Ballou & Lacy, 1995), low breeding rates may occur due to mate incompatibility between individuals in prescribed pairs (Asa, Traylor-Holzer & Lacy, 2011a; Lindburg & Fitch-Snyder, 1994; Martin-Wintle et al., 2015; Quader, 2005). New strategies that incorporate mate choice into conservation efforts are important for enhancing animal productivity and increasing the sustainability of captive populations (Asa, Traylor-Holzer & Lacy, 2011a, 2011b; Lindburg & Fitch-Snyder, 1994; Wedekind, 2002).

Mate choice occurs as a result of non-random allocation of reproductive investment by individuals (Edward, 2015; Paul, 2002). Its mechanisms can be pre-copulatory, whereby visual, chemical, acoustic or behavioral cues influence the likelihood of mating, and/or post-copulatory, whereby copulatory plugs, sperm destruction and other mechanisms alter insemination or fertilization success (reviewed in Neff & Pitcher, 2005; Paul, 2002). Recent literature has demonstrated the importance of the genetic determinants of mate choice in a wide range of species (for an overview of recent publications on this topic, see Table 1). There are currently three main, non-mutually exclusive hypotheses that can explain why the choosier sex (often females) selects mates based on genetic characteristics: (A) quantity of alleles, (B) genetic compatibility between mates, and (C) advantage of particular alleles (reviewed in Kamiya et al., 2014; Setchell & Huchard, 2010). Under the quantity of alleles hypothesis, females experience a fitness advantage by mating with males with greater heterozygosity, or those that carry the greatest number of alleles and hence have the highest genetic diversity (Agbali et al., 2010; Kamiya et al., 2014; Penn & Potts, 1999). Under the genetic compatibility hypothesis, females that mate with males that are genetically dissimilar, or with haplotypes that will best complement the females’, experience a fitness advantage through increased offspring survival or increased offspring genetic diversity (Neff & Pitcher, 2005; Tregenza & Wedell, 2000). Finally, the third hypothesis suggests females prefer males harboring particular alleles that provide offspring with greater immunity to parasites and/or infectious diseases, as often only one or a few alleles provide resistance to a specific pathogen (Bonneaud et al., 2005; Penn & Potts, 1999).

**Table 1:** Summary of MHC-dependent mate choice studies showing the species and MHC genes studied, the hypotheses tested, the study design, and the results for both MHC-dependent mate choice and genome-wide mate choice.

Taxon	Species	MHC class tested	Hypotheses tested*	Study design	Results	Genome-wide testing^†^	Reference
Fish	Atlantic salmon (*Salmo salar*)	Class II	Compatibility	Tested observed MHC mating patterns against randomized mating events	Preference for dissimilar mates	Yes: No effect on mate choice	Landry et al. (2001)
Fish	Atlantic salmon (*Salmo salar*)	Class I	Compatibility	Compared fertilization success of MHC similar and MHC dissimilar pairs	Preference for similar mates	No	Yeates et al. (2009)
Fish	Atlantic salmon (*Salmo salar*)	Class II	Quantity, compatibility and alleles	Compared MHC mating patterns of free mate choice partners against artificial crosses	Preference for dissimilar mates	No	Consuegra & Carlos De Leaniz (2008)
Fish	Brown trout (*Salmo trutta*)	Class II	Compatibility	Tested observed MHC mating patterns against randomized mating events	Preference for intermediate similarity	Yes: No effect on mate choice	Forsberg et al. (2007)
Fish	Broadnosed pipefish (*Syngnathus typhle*)	Class I	Compatibility	Compared MHC of preferred partners with non-preferred partners	Preference for dissimilar mates	Yes: No effect on mate choice	Roth et al. (2014)
Fish	Chinook salmon (*Oncorhynchus tshawytscha*)	Class II	Quantity and compatibility	Tested observed MHC mating patterns against randomized mating events	Preference for dissimilar mates	Yes: No effect on mate choice	Neff et al. (2008)
Fish	Three-spined stickleback (*Gasterosteus aculeatus*)	Class II	Quantity, compatibility and alleles	Tested observed MHC mating patterns against randomized mating events	Preference for certain alleles and intermediate similarity	Yes: No effect on mate choice	Eizaguirre et al. (2009)
Amphibian	Tiger salamander (*Ambystoma tigrinum*)	Class II	Compatibility	Compared reproductive success of MHC similar matings with MHC dissimilar matings	Preference for similar mates	Yes: No effect on mate choice	Bos et al. (2009)
Reptile	Sand Lizard (*Lacerta agilis*)	Class I	Quantity and compatibility	Compared MHC of preferred partners with non-preferred partners in the laboratory and tested observed MHC mating patterns against randomized mating events in the field	Preference for dissimilar mates in the laboratory and field	No	Olsson et al. (1996)
Reptile	Tuatara (*Sphenodon punctatus*)	Class I	Quantity and compatibility	Tested observed MHC mating patterns against randomized mating events	Preference for dissimilar mates	Yes: No effect on mate choice	Miller et al. (2009)
Bird	House sparrow (*Passer domesticus*)	Class I	Quantity, compatibility and alleles	Compared MHC of preferred partners with non-preferred partners	Preference for diverse mates and similar mates	Yes: No effect on mate choice	Bonneaud et al. (2006)
Bird	Rose bitterling (*Rhodeus ocellatus*)	Class II	Compatibility	Compared MHC of preferred partners with non-preferred partners	Preference for dissimilar mates	No	Agbali et al. (2010)
Bird	Seychelles warbler (*Acrocephalus sechellensis*)	Class I	Quantity and compatibility	Tested observed MHC mating patterns against randomized mating events and compared the occurrence of extra-pair paternity with MHC of social and extra-pair pairs	No preference for social mates but greater occurrence of extra-pair paternity when social pairs have low MHC diversity and when extra-pair males have higher diversity	Yes: No effect on mate choice	Richardson et al. (2005)
Mammal	Alpine marmot (*Marmota marmota*)	Class I and Class II	Quantity, compatibility and alleles	Tested observed MHC mating patterns against randomized mating events and compared the occurrence of extra-pair paternity and number of extra-pair young with MHC of social pairs	Preference for dissimilar social mates at MHCII loci and greater occurrence of extra-pair paternity when social pairs have low MHCII dissimilarity	Yes: No effect on social mate choice	Ferrandiz-Rovira et al. (2016)
Mammal	Bank vole (*Clethrionomys glareolus*)	Class II	Quantity and compatibility	Compared MHC of preferred partners with non-preferred partners	Preference for dissimilar mates	No	Radwan, Tkacz & Kloch (2008)
Mammal	Chacma baboon (*Papio ursinus*)	Class II	Quantity, compatibility and alleles	Tested observed MHC mating patterns against randomized mating events	No influence of MHC on mate choice	Yes: No effect on mate choice	Huchard et al. (2010)
Mammal	Fat-tailed Dwarf lemur (*Cheirogaleus medius*)	Class II	Quantity, compatibility and alleles	Tested observed MHC mating patterns against randomized mating events and compared the occurrence of extra-pair paternity with MHC of social pairs	Preference for diverse and dissimilar mates and greater occurrence of extra-pair paternity when social pairs have low MHC dissimilarity	Yes: Preference for more diverse mates	Schwensow et al. (2008)
Mammal	Gray mouse lemur (*Microcebus murinus*)	Class II	Quantity, compatibility and alleles	Tested observed MHC mating patterns against randomized mating events	Preference for diverse and dissimilar mates	Yes: Preference for more diverse mates	Schwensow, Eberle & Sommer (2008)
Mammal	Gray mouse lemur (*Microcebus murinus*)	Class II	Quantity and compatibility	Tested observed MHC mating patterns against randomized mating events	Preference for dissimilar mates at 1 locus	Yes: Preference for less related individuals	Huchard et al. (2013)
Mammal	Malagasy giant jumping rat (*Hypogeomys antimena)*	Class II	Compatibility	Tested observed MHC mating patterns against randomized mating events	Preference for similar mates	No	Sommer (2005)
Mammal	Mandrill (*Mandrillus sphinx)*	Class II	Quantity, compatibility and alleles	Compared MHC of sires with non-sires	Preference for diverse and dissimilar mates	Yes: Preference for less related and dissimilar mates	Setchell et al. (2010)
Mammal	Rhesus Macaque (*Macaca mulatta*)	Class II	Quantity, compatibility and alleles	Compared MHC of sires with non-sires and tested observed MHC mating patterns against randomized mating events	Preference for diverse mates	Yes: No effect on mate choice	Sauermann et al. (2001)
Mammal	Soay sheep (*Ovis aries*)	Class I and Class II	Compatibility	Tested observed MHC mating patterns against randomized mating events	No influence of MHC on mate choice	Yes: No effect on mate choice	Paterson & Pemberton (1997)
Mammal	Tuco-tuco (*Ctenomys talarum*)	Class II	Quantity, compatibility and alleles	Compared MHC of preferred partners with non-preferred partners in the laboratory and tested observed MHC mating patterns against randomized mating events in the field	Preference for certain alleles in the laboratory and preference for diversity and certain alleles in the field	No	Cutrera, Fanjul & Zenuto (2012)

**Notes:**

* Quantity = quantity of MHC alleles hypothesis; Compatibility = genetic compatibility hypothesis; Alleles = advantage of particular alleles hypothesis.

† Testing whether genome-wide characteristics, such as heterozygosity at non-MHC markers, influenced mate choice decisions.

These hypotheses may apply genome-wide, or to specific loci, such as genes of the major histocompatibility complex (MHC). It is well known that MHC genes play a vital role in the vertebrate adaptive immune response (Balakrishnan & Adams, 1995). Within the MHC gene family there are two main classes of molecules: class I molecules bind virus-derived peptides, and class II molecules bind peptides from extracellular bacteria and larger parasites (Balakrishnan & Adams, 1995; Milinski, 2006). Therefore, having many different MHC alleles increases the ability to respond to a larger range of pathogens (Penn & Potts, 1999). We focused on classical class II MHC genes due to their purported role in mammalian mate choice (Table 1).

Numerous studies have found evidence for one or more of the proposed hypotheses, considering both genome-wide and MHC-dependent mate preferences (Table 1). Fish and reptiles often show a preference for mates that are more dissimilar at MHC loci, in accordance with the genetic compatibility hypothesis. Birds and mammals have been found to show preferences for both diverse and dissimilar mates (under the quantity of alleles and genetic compatibility hypotheses respectively) (Table 1). In other instances, fish and mammal species show preferences for specific MHC alleles in line with the advantage of particular alleles hypothesis (Eizaguirre et al., 2009; Cutrera, Fanjul & Zenuto, 2012). Interestingly, many studies have examined the influence of genome-wide diversity on mate choice preferences. In mammalian species there is a preference for mates that are more diverse or dissimilar overall (Table 1). These studies have been crucial in demonstrating the importance of genetic determinants of mate choice; however, it is also important to consider other, non-genetic factors that may underlie mate choice decisions.

**Figure 1:**
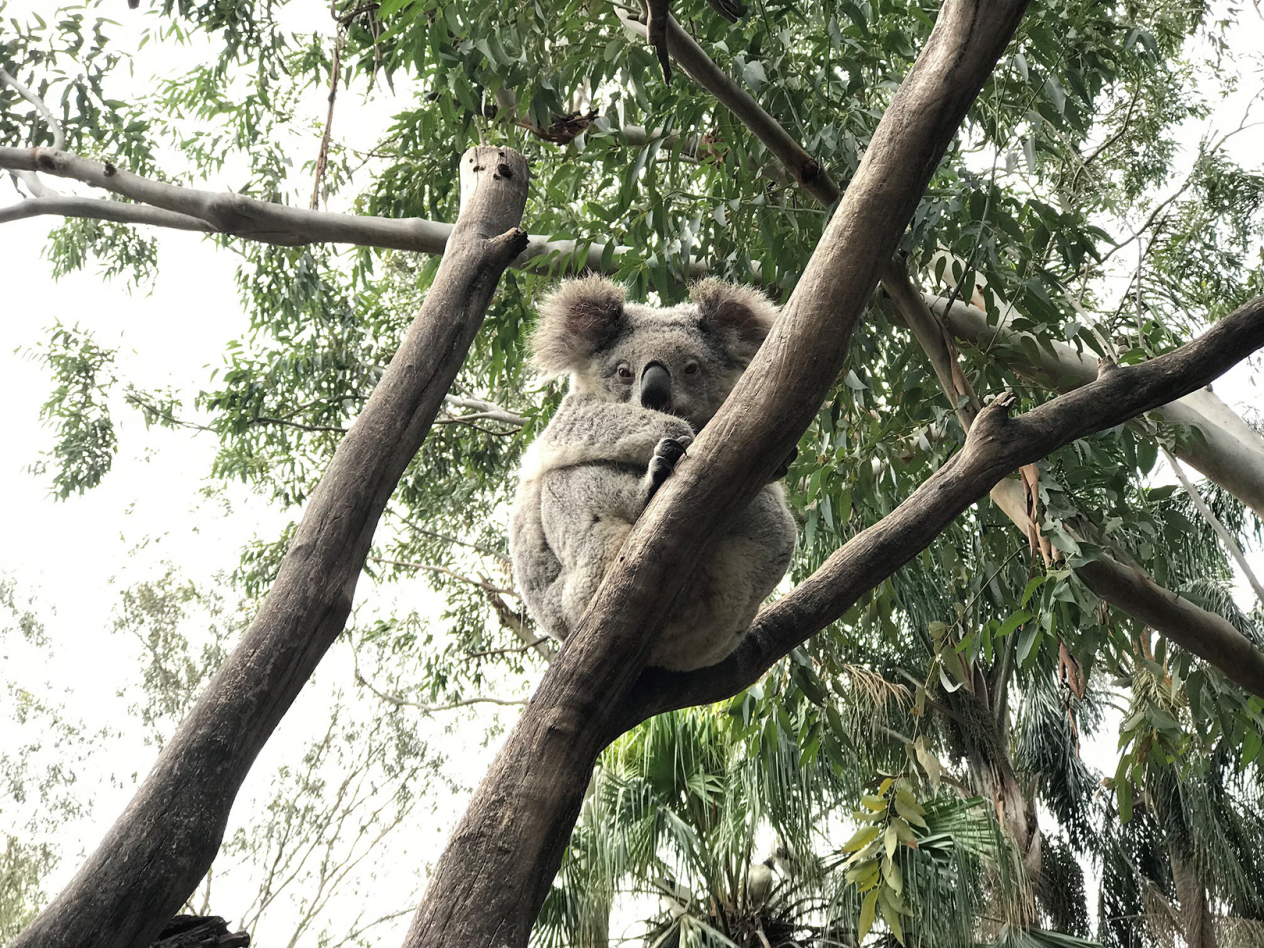
The koala (*Phascolarctos cinereus*): an arboreal folivorous marsupial. Photo credit: Parice Brandies.

**Figure 2:**
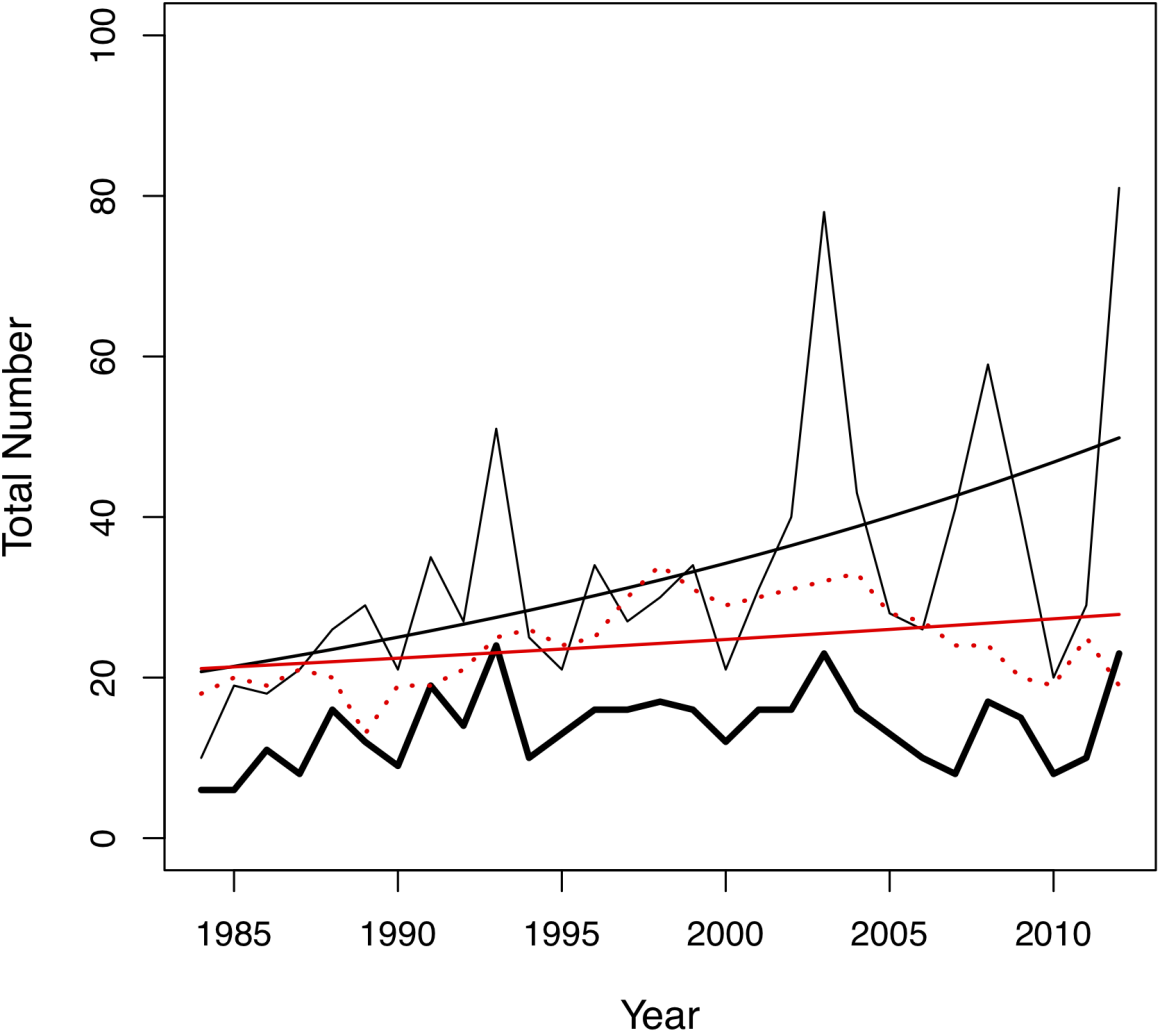
Changes in the total number of pairing events (thin black line), unique male-female pair combinations (thick black line) and individuals in the colony (red dotted line) per year in the San Diego Zoo koala population over time (*n* = 29 years). Changes were modelled using generalized linear models (GLMs) with Poisson distribution. A trend line is plotted for the total number of pairing events (ß = 0.03, SE = 0.004, *p* < 0.001, black line) and total number of individuals in the colony (ß = 0.01, SE = 0.005, *p* = 0.028, red line).

**Figure 3:**
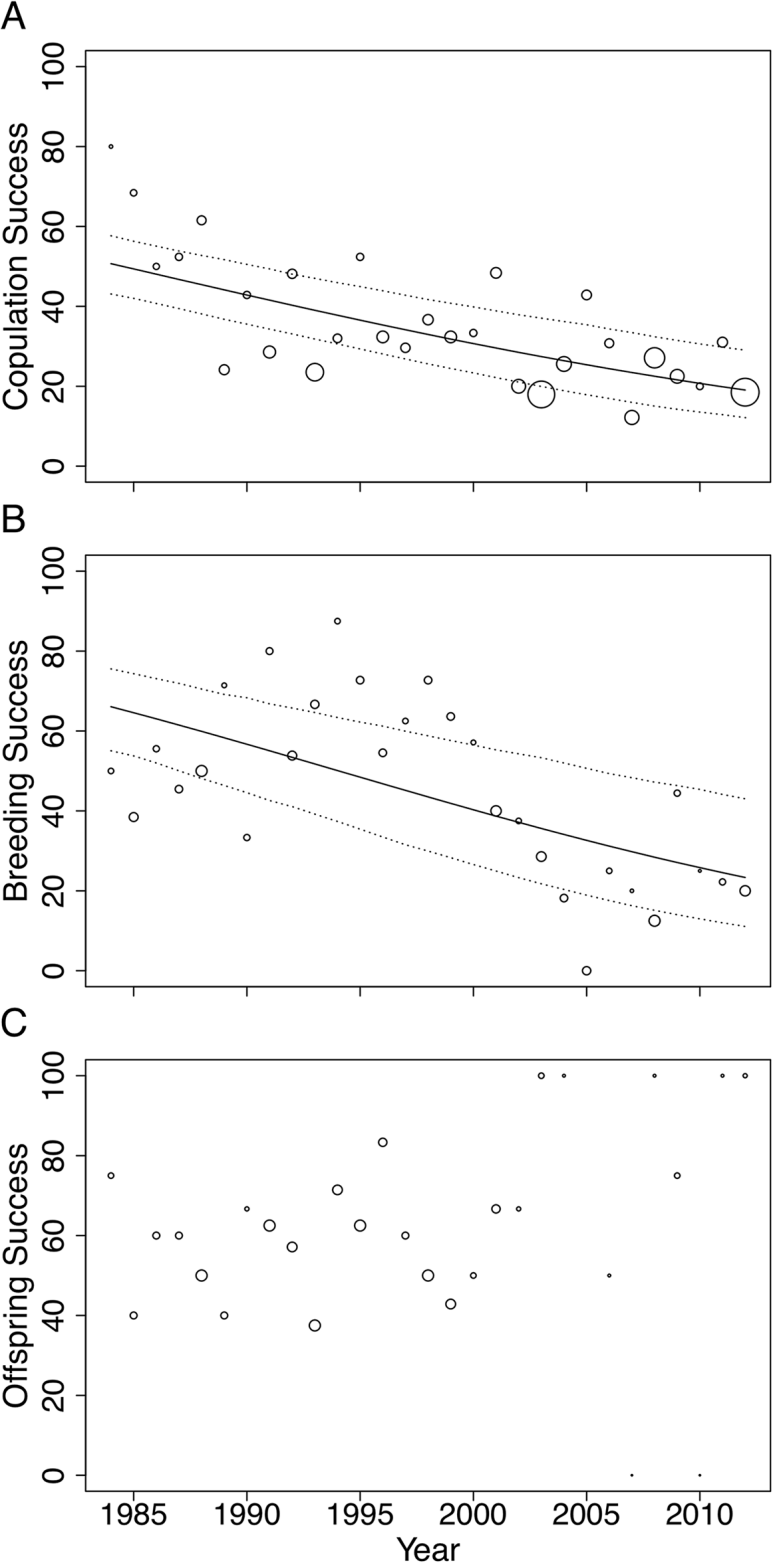
Changes in (A) copulation success, (B) breeding success and (C) offspring success rates of the San Diego Zoo koala population over time (*n* = 29 years). Changes were modelled using GLMs with binomial distribution. Trend lines are plotted for copulation success (ß = −0.05, SE = 0.009, *p* < 0.001) and breeding success (ß = −0.07, SE = 0.015, *p* < 0.001). Dotted lines represent 95% CI obtained by parametric bootstrapping of the intercept and slope. Point size correlates to number of pairings, number of copulations and number of offspring in (A), (B) and (C), respectively.

In this study, we investigated the role of mate choice in the San Diego Zoo koala colony (Fig. 1). San Diego Zoo’s breeding program commenced in 1981, and houses the largest koala colony outside of Australia. As a managed zoo population, koalas are bred using a mean kinship strategy, restricting free access to mates. Despite increased pairing efforts (Fig. 2), the colony has shown significant declines in copulation and breeding success over time (Fig. 3). Familiarity and age have previously been proposed to be important factors involved in captive koala mate choice (Bercovitch et al., 2006), while evidence for size-mediated sexual selection in the koala is contradictory due to variable results (Bercovitch et al., 2006; William & Bercovitch, 2011). The vomeronasal organ of the koala is predicted to play a role in MHC-based olfactory discrimination (Hegde, 2003) and suggests a potential mechanism for this species to select mates based on genetic characteristics in natural settings.

In other species, studies of MHC-dependent mate choice have employed MHC typing techniques to examine links between MHC genes and mate choice patterns (Huchard et al., 2010). However, due to the large numbers of duplicated MHC loci throughout marsupial genomes (Belov et al., 2013; Nei, Gu & Sitnikova, 1997), obtaining genotypes for individual koalas at multiple MHC loci using current MHC typing methods is impractical. Furthermore, a recent meta-analysis revealed that a multi-locus approach is necessary for testing MHC-dependent mate choice associations (Kamiya et al., 2014). MHC-linked microsatellites have been shown to be good proxies for variation at MHC loci and are popular as an accurate, fast and cost-effective alternative to genotyping individuals at multiple MHC loci (Cheng & Belov, 2012; Cheng et al., 2009a; Crouau-Roy et al., 1996). The recent characterization of MHC genes in the koala genome (Johnson et al., 2018) enables specific MHC-linked microsatellites to be identified in variable, single-copy regions, overcoming the difficulties of previous MHC typing approaches. Here, we employ MHC-linked microsatellites to quantify diversity at MHC and non-MHC-linked microsatellites to quantify genome-wide diversity (Roth et al., 2014). Determining how species make mate choice decisions, and the extent to which both non-genetic and genetic factors influence breeding success in captive populations, will enable more effective captive breeding strategies and assist in improving the sustainability of captive breeding programs (Asa, Traylor-Holzer & Lacy, 2011a; Quader, 2005).

Our study aims to (1) investigate non-genetic factors (such as age and familiarity) that may influence mate choice in captive koalas colony using detailed pairing records; and (2) test the three mate choice hypotheses (quantity of alleles, genetic compatibility and advantage of particular alleles) in regard to both MHC-dependent (using MHC-linked microsatellites) and genome-wide (using non-MHC-linked microsatellites) mating preferences.

## Methods

### Study samples

A total of 70 koala DNA samples were extracted from banked whole blood and tissue samples (previously collected under San Diego Zoo Global IACUC protocols 10-008, 10-009, 11-029, 14-034) using either standard phenol-chloroform extraction, or the QIAamp DNA Mini Kit (Qiagen, Hilden, Germany) following manufacturer’s directions. Detailed pairing records, and studbook data, were provided by the Association of Zoos and Aquariums North American Regional Studbook Keeper (Chris Hamlin, personal communication, April 2017). These pairing records spanned 1984–2012 and contained mate choice data and breeding outcomes for every pairing (*n* = 964) at the zoo throughout this period. Breeding recommendations are reviewed annually, and predominately follow a minimize kinship strategy (Ballou & Lacy, 1995). During pairing, oestrous females are placed with males in enclosed cubicles for 5–10 min and mating behavior is monitored throughout this time (Bercovitch et al., 2006). If the pair does not copulate, the female and male may be paired with other conspecifics, or the same pairing may be trialed again at a later time. This pairing process means both female and male koalas are often exposed to multiple individuals of the opposite sex within and between seasons. We are therefore able to determine whether mate choice is occurring at the pre-copulatory level (i.e., copulation success as a proportion of pairing attempts) or post-copulatory level (i.e., breeding success (production of offspring) as a proportion of copulations), and whether it affects offspring success (offspring that survive more than 1 year as a proportion of offspring produced).

DNA samples were available for individuals across 28 years of pairing data (Fig. S1), accounting for 51% of the individuals and 49% of the pairings in the complete dataset (Table S1). A total of 22 sampled koalas were never paired for breeding and were excluded from the final mate choice analyses. These koalas were genotyped during the study and included in marker diversity statistics (see “Marker diversity” in the Supplemental Information) to maximize sample sizes where possible. All non-genetic factors were analyzed using the complete pairing dataset (including unsampled koalas).

### Non-genetic determinants of koala mate choice

We used generalized linear models (GLMs) in R v 3.4.0 (R Core Team, 2017) to test for non-genetic effects, including year, age and familiarity, on mating success (copulation, breeding and offspring success; see below). GLMs were performed with binomial distribution as follows: for each pairing event (*n* = 964) we modelled whether the pair successfully copulated (1) or did not copulate (0), with predictor variables including year of the pairing (to account for changes in the breeding program over time), age of the female, age of the male and the number of years the male and female had previously been paired together (as a measure of familiarity). Age^2^ was also included, as the relationship between age and mating success was not predicted to be linear (Rose, 1991). The dataset was then subset into only those pairs that successfully copulated (*n* = 304) and the same predictor variables were modelled against whether each of these pairs successfully bred (produced offspring) (1) or did not breed (0). The dataset was then further subdivided into only those pairs that successfully bred (*n* = 134) and the same predictor variables were modelled against whether those pairs produced offspring that survived more than 1 year (1) or did not survive more than 1 year (0).

Male body mass is a strong predictor of male koala breeding success in the wild (William & Bercovitch, 2011) and correlates strongly with male age (Tobey et al., 2006). We did not have male body mass available for our study animals. As age difference between the male and female was also highly correlated with both female and male age (ρ = −0.58, *p* = < 0.001 and ρ = 0.76, *p* = < 0.001, respectively, Table S2) it was not included in the model. As a result, any effects of male age that we observe may also be reflecting the effect of male body mass and/or the age difference of the pair; female age effects may also be driven by age difference.

Although some pairs were repeated in multiple years, Pair ID was not included as a random factor due to the majority of pairs (60%) only being represented in 1 year of the dataset (Fig. S2) (models with Pair ID fitted as a random intercept did not converge). Variance inflation factors (VIFs; Belsley, Kuh & Welsch, 1980) were calculated for the remaining predictor variables to ensure there were no adverse effects of multicollinearity. All VIFs were <2 and so year, female age, male age and familiarity were included in the models (Belsley, Kuh & Welsch, 1980). Model predictors were standardized by subtracting the mean and dividing by two standard deviations (following Gelman, 2008) to facilitate inference of regression coefficients within and between models (Schielzeth, 2010). Model fitted values were back-transformed onto the natural scale for plotting and interpretation.

### MHC genotyping

Major histocompatibility complex-linked primers were designed within 10 kb of MHC Class II genes that had been annotated by the Koala Genome Consortium (Johnson et al., 2018). RepeatMasker (Smit, Hubley & Green, 2013–2015) was used to identify microsatellite sequences <10 kb away from the MHC genes (Cheng et al., 2009b). Candidate microsatellite sequences (PhciDBB001M3, PhciDCBM1 and MHCIIDAB001M1) were selected based on minimal interruptions to the repeat sequence and low proximity to other repeat regions. These microsatellites were linked to genes of the DB, DC and DA families respectively (Abts, Ivy & DeWoody, 2015; Lau et al., 2013, 2014a; Lau, Griffith & Higgins, 2014b), allowing us to incorporate a representative for each classical marsupial MHC class II family (Belov et al., 2006; Belov, Lam & Colgan, 2004). The repeat motifs for each microsatellite were (TG)_13_, (GA)_28_ and (AC)_29_ respectively. We extracted these microsatellite sequences with 300 bp of flanking sequence and designed PCR primers using Oligo 7 (Rychlik, 2007). Primer sequences were then used in a BLAST search (Priyam et al., 2015) against the koala genome (Johnson et al., 2018) to ensure specificity and prevent amplification of non-target sequences. Primer sequences used to amplify the three microsatellites were as follows (5′ to 3′): PhciDBB001M3 F:TTCTCTTGTCCTTCTTGTGTC, R:TTCTCCCTACAAAGATGATCC; PhciDCBM1 F:AGTCTGGTGTCATTAGCAATAGG, R:CTGAATGAGGCAAGGGAGAG; MHCIIDAB001M1 F:ACACTACTTCCCTGAATCTGAC, R:TACAGTGTTACTTCATGCAGAG.

All loci were initially screened for polymorphism using DNA samples from koalas previously found to be polymorphic at MHC loci (Cheng et al., 2017) (see “Initial primer screening and optimization methods” in the Supplemental Information) before genotyping the study population at these markers. Since all three markers had similar product lengths, they were not multiplexed for typing. PCRs were carried out for each locus using the Type-it Microsatellite PCR Kit (Qiagen, Hilden, Germany) with a modified total reaction size of 10 μL and the following modified primer concentrations: 0.06 μM tagged primer, 0.6 μM untagged primer and 0.6 μM 6-FAM labelled CAG tag (Schable, Fischer & Glenn, 2002) (see Table S3 for thermocycling conditions). Capillary electrophoresis was undertaken at the Australian Genome Research Facility using MCLAB DSMO-100 Orange Size Standard. Alleles were manually called using GeneMarker (Hulce et al., 2011). Controls included a negative control using water, and a positive control using DNA from a koala that was successfully genotyped during the initial primer development.

### Genotyping of non-MHC-linked microsatellites

Koalas were genotyped at a further 15 microsatellite loci, not known to be linked to MHC, using primers from previous studies (Cristescu et al., 2009; Dennison et al., 2017; Houlden, England & Sherwin, 1996) (Table S4). The genomic location of each microsatellite was confirmed using the NCBI koala assembly browser (Kitts et al., 2015). All microsatellites were located on scaffolds not containing any MHC genes, and seven were >10 kb away from any genes. Seven markers (Pcin05, Pcin08, Pcin11, Pcin20, Pcin21, Pcin22 and Pcin23) were split into three multiplexes and typed using a fluorescently labelled (6-FAM) CAG-tag (Schable, Fischer & Glenn, 2002) (Table S4), with PCRs carried out using the Type-it Microsatellite PCR Kit (Qiagen, Hilden, Germany) (as above). The remaining eight markers were amplified separately using a fluorescently labelled (HEX or 6-FAM) M13 tail (0.6 μM) (Schuelke, 2000) or forward primer (Table S4). PCRs were carried out in a 10 μL reaction volume containing 1× PCR buffer, 2.5 mM MgCl, 0.2 mM dNTPs (Apex; Genesee Scientific, San Deigo, CA, USA), 0.2 μM forward primer, 0.6 μM reverse primer and 0.5 U AmpliTaq Gold (Applied Biosystems, Waltham, MA, USA) (see Table S3 for thermocycling conditions). Samples were genotyped on an ABI 3130xl using an internal GeneScan 500 ROX size standard and alleles were automatically called then manually checked using GeneMapper (ABI).

### Microsatellite diversity

Approximately 20% of the koalas were re-genotyped to determine genotyping error rate. Tests for evidence of null alleles, deviation from Hardy–Weinberg equilibrium, and linkage disequilibrium were performed to ensure all of the non-MHC and MHC markers were suitable for use in the final statistical analysis (Methods and Results for these analyses are provided in “Marker diversity” in the Supplemental Information and Table S5). Standardized heterozygosity (H_s_) was calculated as a measure of individual multilocus heterozygosity at both the non-MHC and MHC markers using the Rhh package (Alho, Välimäki & Merilä, 2010) in R. We chose this method, as H_s_ gives equal weighting to all loci examined despite variation in the number and frequency of alleles present across the markers used (Aparicio, Ortego & Cordero, 2006; Coltman et al., 1999). A Spearman’s rank correlation between standardized heterozygosity at MHC-linked markers and standardized heterozygosity at non-MHC markers was also performed to test whether MHC diversity and genome-wide diversity were correlated. This was necessary to determine whether any MHC-associated mate choice findings were by-products of genome-wide variation (Ferrandiz-Rovira et al., 2016).

### Statistical analysis

GLMs were used to test the three mate choice hypotheses at pre-copulatory, post-copulatory and offspring survival stages using the three binomial response variables: copulation success, breeding success and offspring success respectively (defined above). In each model, the predictor variables (male heterozygosity, pair similarity and allele presence/absence) were as described in the sections that follow. Multicollinearity of predictor variables in all models was checked by calculating VIFs, and model predictors were standardized to facilitate inference across predictors, as described above.

#### Quantity of alleles

To test whether mating success was influenced by genome-wide quantity of alleles, male standardized heterozygosity (H_s_) (non-MHC or MHC markers) was modelled as the predictor, with copulation, breeding and offspring success for each male as separate response variables. For MHC, we also tested heterozygosity each locus individually (coded 1/0 for heterozygote/homozygote). Year of first pairing for each male was included to account for changes in the program over time (year of first pairing was highly correlated with year; ρ = 0.98, *p* = < 0.01). For copulation models, male age at first pairing was also included as a predictor to account for the influence of male age on copulation success (see Results).

#### Genetic compatibility

To test whether genome-wide genetic compatibility influenced mating success, we modelled molecular coancestry (allele sharing, evaluated using MolKin v 2.0; Gutiérrez et al., 2005) as a predictor of copulation, breeding or offspring success. We used molecular coancestry, because meaningful estimates of allele frequencies are difficult to calculate for captive populations with complex pedigrees and managed mating strategies (Ivy et al., 2016). Pairwise similarity at MHC loci was calculated using Wetton’s formula (Parkin et al., 1987): D_AB_ = 2F_AB_/(F_A_+F_B_); where, F_AB_ is the total number of unique MHC-linked microsatellite alleles shared by a male (A) and a female (B) across the typed loci; and F_A_ and F_B_ are, respectively, the total number of alleles of the male (A) and female (B) (Parkin et al., 1987). This formula is commonly used to determine similarity at MHC loci (Huchard et al., 2010; Olsson et al., 2003) and enabled us to assess each MHC locus (DA, DB and DC) separately (other similarity estimators rely on multi-locus data). The first year of pairing for each pair was added to all models as an additional predictor. For copulation models, male age at first pairing and the total number of years paired together were also included.

#### Advantage of particular alleles

Under the advantage of the particular alleles hypothesis, the null hypothesis that specific MHC alleles do not influence mating success was tested by coding each male with a 1/0 predictor indicating the presence or absence, respectively, of each allele of the three MHC-linked loci (Sepil et al., 2012); and modelling these predictors separately against each of the response variables of copulation, breeding and offspring success. Year of first pairing was included as a predictor in every model; and male age at first pairing was also included in the copulation models. A “base” model, which excluded allelic information, was fitted for each response variable across the three loci. For each response variable, models were ranked by AIC_C_ (Burnham & Anderson, 2002) to determine the relative level of support for each allele as a predictor of mating success. Models that were highly ranked (i.e., ≥2 AIC_C_ above the next best model and above the base model) were interpreted as providing strong evidence that the presence or absence of a given allele had an effect on the corresponding response variable (Sepil et al., 2012).

## Results

### Non-genetic determinants of koala mate choice

Male age had a significant effect on copulation success (Table 2). Expected copulation success rates increased from ∼20% when males were 2-years-old, to 40% when males reached 12 years of age, and decreased to below 35% when males were 17 years or older (fitted values are taken from the regression model in Table 2). Copulation success increased significantly with increasing familiarity between pairs (Table 2). Dyads that had previously been paired together for 5 years or more had expected copulation success rates above 50% (95% CI [0.36–0.65]), compared to the 34% success rate of dyads that had never been paired (95% CI [0.28–0.41]; fitted values are taken from the regression model in Table 2). No association was found between female age and mating success (Table 2). None of the factors we tested influenced breeding or offspring success and could not explain the strong declines in copulation and breeding success across time (Table 2).

**Table 2:** Generalized linear models of the relationships between year, age, familiarity and three measures of mating success.

Response variable	*n*	Predictor variable	Slope ± SE	*z*-value	*p*
Copulation success	964	**Year**	**−0.97 ± 0.16**	**−6.083**	**<0.001**
		Female age^2^*	−0.53 ± 0.29	−1.866	0.062
		Female age	−0.30 ± 0.18	−1.728	0.084
		Male age^2^*	**−0.67 ± 0.29**	**−2.266**	**0.023**
		**Male age**	**0.54 ± 0.20**	**2.713**	**0.007**
		**Familiarity**	**0.34 ± 0.17**	**2.031**	**0.042**
Breeding success	304	**Year**	**−1.07 ± 0.29**	**−3.729**	**<0.001**
		Female age^2^*	0.10 ± 0.41	0.244	0.807
		Female age	−0.30 ± 0.30	−1.024	0.306
		Male age^2^*	0.37 ± 0.46	0.802	0.422
		Male age	−0.30 ± 0.31	−0.963	0.335
		Familiarity	0.05 ± 0.29	0.185	0.853
Offspring success	134	**Year**	**0.89 ± 0.45**	**2.007**	**0.045**
		Female age^2^*	−0.70 ± 0.62	−1.127	0.260
		Female age	−0.31 ± 0.44	−0.713	0.476
		Male age^2^*	−0.02 ± 0.64	−0.038	0.970
		Male age	−0.33 ± 0.50	−0.653	0.514
		Familiarity	0.64 ± 0.49	1.299	0.194

**Notes:**

Predictor variables were standardized by subtracting the mean and dividing by two standard deviations (see Methods).

Predictors in bold show coefficients that are statistically different from 0 at the 0.05 α level.

* Squared term used to create a polynomial model as the relationship between age and mating success was not predicted to be linear.

### Microsatellite genotyping

All koalas were genotyped at the three MHC markers and >75% of the study population was genotyped at 13 or more of the non-MHC loci (Table S5). Genotyping error rate was very low (0.53%). For the MHC-linked markers, the mean number of alleles per locus (N_a_) was 9.67 (range 7–13; *n* = 3 loci, 70 koalas), mean observed heterozygosity (H_o_) was 0.771 ± 0.1 (SE), and mean expected heterozygosity (H_e_) was 0.755 ± 0.083 (Table S5). Standardized heterozygosity (H_s_) for the MHC markers ranged from 0.432 (more homozygous) to 1.3 (more heterozygous) (Table S5). Non-MHC marker H_s_ ranged from 0.347 (more homozygous) to 1.62 (more heterozygous). For the non-MHC markers, N_a_ = 6.53 (range 2–10; *n* = 15 loci, 70 koalas), H_o_ = 0.673 ± 0.05 and H_e_ = 0.662 ± 0.046 (Table S5). Standardized heterozygosity at MHC-linked loci was not correlated with standardized heterozygosity at non-MHC loci (*n* = 70 koalas, ρ = −0.06, 95% CI [−0.30–0.19], *p* = 0.614).

### Genetic determinants of koala mate choice

#### Quantity of alleles

For MHC-linked loci, there was a negative relationship between male H_s_ and copulation success (Table 3A). Examining each MHC locus separately suggested that the overall trend may result primarily from heterozygosity at the MHCII DAB locus (Table 3B). Amongst those males that successfully copulated, males with higher overall MHC H_s_ showed significantly greater breeding success rates than less heterozygous males (Table 3A). For example, our models predict that males that were heterozygous at all three MHC-linked loci had expected copulation success rates of 22% (95% CI [0.15–0.31]) and breeding success rates of 49% (95% CI [0.29–0.68]), whereas males that were homozygous at all three MHC-linked loci had expected copulation success rates of 56% (95% CI [0.34–0.77]) and breeding success rates of 9% (95% CI [0.02–0.32]; fitted values are taken from the regression models in Table 3). No association was found between offspring survival and male H_s_ at MHC loci (Table 3). For non-MHC-linked loci, male H_s_ did not show a significant effect on copulation, breeding or offspring success (Table 3C). Year and age had a significant influence on mating success (Table 3).

**Table 3:** Generalized linear models of the relationship between male heterozygosity and mating success.

Response variable	*n*	Predictor variable*	Slope ± SE	*z*-value	*p*
A. Overall MHC heterozygosity					
Copulation success	21	**Intercept**	**−0.79 ± 0.102**	**−7.74**	**<0.001**
		**Year**	**−1.32 ± 0.230**	**−5.77**	**<0.001**
		**Age**	**0.46 ± 0.169**	**2.71**	**0.007**
		**H_s_**	**−0.51 ± 0.217**	**−2.35**	**0.019**
Breeding success	17	**Intercept**	**−0.77 ± 0.201**	**−3.83**	**<0.001**
		**Year**	**−1.51 ± 0.414**	**−3.65**	**<0.001**
		**H_s_**	**0.79 ± 0.372**	**2.13**	**0.034**
Offspring success	13	**Intercept**	**1.35 ± 0.493**	**2.73**	**0.006**
		Year	1.68 ± 0.925	1.82	0.069
		H_s_	−0.76 ± 0.687	−1.10	0.270
B. Individual MHC heterozygosity					
Copulation success	21	**Intercept**	**−0.79 ± 0.102**	**−7.77**	**<0.001**
		**Year**	**−1.48 ± 0.279**	**−5.30**	**<0.001**
		**Age**	**0.46 ± 0.169**	**2.75**	**0.006**
		DBB heterozygosity (6,15)	−0.31 ± 0.239	−1.32	0.187
		DCB heterozygosity (3,18)	−0.37 ± 0.341	−1.10	0.273
		**DAB heterozygosity (4, 17)**	**−0.73 ± 0.346**	**−2.10**	**0.036**
Breeding success	17	**Intercept**	**−0.79 ± 0.211**	**−3.76**	**<0.001**
		**Year**	**−1.40 ± 0.489**	**−2.86**	**0.004**
		DBB heterozygosity (5, 12)	0.55 ± 0.379	1.44	0.149
		DCB heterozygosity (2, 15)	0.82 ± 0.563	1.45	0.147
		DAB heterozygosity (3, 14)	0.97 ± 0.846	1.14	0.253
Offspring success	13	**Intercept**	**1.26 ± 0.402**	**3.13**	**0.002**
		**Year**	**1.83 ± 0.881**	**2.07**	**0.038**
		DBB heterozygosity (4, 12)	−1.05 ± 0.625	−1.69	0.092
		DCB heterozygosity (1, 12)	NA	NA	NA
		DAB heterozygosity (1, 12)	NA	NA	NA
C. Genome-wide heterozygosity					
Copulation success	21	**Intercept**	**−0.91 ± 0.094**	**−9.75**	**<0.001**
		**Year**	**−1.69 ± 0.334**	**−5.07**	**<0.001**
		**Age**	**0.37 ± 0.163**	**2.28**	**0.023**
		H_s_	−0.47 ± 0.324	−1.45	0.146
Breeding success	17	**Intercept**	**−0.59 ± 0.178**	**−3.32**	**0.001**
		**Year**	**−1.00 ± 0.495**	**−2.03**	**0.042**
		H_s_	0.62 ± 0.512	1.22	0.224
Offspring success	13	**Intercept**	**0.98 ± 0.353**	**2.79**	**0.005**
		Year	1.26 ± 0.792	1.59	0.112
		H_s_	0.24 ± 0.887	0.27	0.788

**Notes:**

Predictor variables were standardized by subtracting the mean and dividing by two standard deviations (see Methods).

Predictors in bold show coefficients that are statistically different from 0 at the 0.05 α level.

* Numbers in parentheses indicate the number of homozygotes and heterozygotes respectively. Any loci with <2 homozygotes were not fitted but are shown in the table for completeness (denoted “NA”).

#### Genetic compatibility

Similarity at MHC-linked loci did not have a significant effect on copulation success (Table 4); however, pairs with a higher similarity at the MHCII DAB-linked locus had a significantly greater breeding success rate than more dissimilar pairs (Table 4B). For example, our models predicted that pairs that share one or more alleles at the MHCII DAB locus would have an expected breeding success rate of 40% or higher (95% CI [0.24–0.56]), compared to pairs that shared no alleles, which would have an expected breeding success rate of 20% (95% CI [0.13–0.30]; fitted values are taken from the regression model in Table 4). There were no significant effects of pairwise MHC similarity on offspring success (Table 4). Genome-wide (non-MHC) similarity did not have a significant effect on copulation nor offspring success; however, pairs with a higher similarity at non-MHC loci had significantly greater breeding success rates than more dissimilar pairs (Table 4C). For example, expected breeding success rates increased from 15% (95% CI [0.08–0.27]) to 57% (95% CI [0.24–0.84]) as genome-wide similarity estimates increased from 0.2 (low allele sharing at non-MHC loci between pairs) to 0.6 (high allele sharing at non-MHC loci between pairs) respectively (fitted values are taken from the regression model in Table 4). Year, familiarity and male age were all found to have the same effects on mating success as above (Table 4).

**Table 4:** Generalized linear models of the relationship between pair similarity and mating success.

Response variable	*n*	Predictor variable	Slope ± SE	*z*-value	*p*
A. Overall MHC Similarity					
Copulation success	89	**Intercept**	**−1.16 ± 0.132**	**−8.79**	**<0.001**
		**Year**	**−0.60 ± 0.242**	**−2.46**	**0.014**
		**Familiarity**	**0.46 ± 0.212**	**2.17**	**0.030**
		Male age	0.44 ± 0.241	1.84	0.066
		MHC similarity	0.32 ± 0.201	1.59	0.112
Breeding success	53	**Intercept**	**−1.02 ± 0.223**	**−4.59**	**<0.001**
		**Year**	**−1.31 ± 0.427**	**−3.08**	**0.002**
		MHC similarity	0.74 ± 0.382	1.93	0.054
Offspring success	26	**Intercept**	**1.42 ± 0.542**	**2.62**	**0.009**
		Year	1.40 ± 0.847	1.66	0.098
		MHC similarity	0.40 ± 0.701	0.56	0.572
B. Individual MHC similarity					
Copulation success	89	**Intercept**	**−1.16 ± 0.133**	**−8.72**	**<0.001**
		**Year**	**−0.71 ± 0.255**	**−2.77**	**0.006**
		**Familiarity**	**0.48 ± 0.212**	**2.28**	**0.022**
		**Male age**	**0.51 ± 0.245**	**2.08**	**0.038**
		DBB similarity	0.44 ± 0.227	1.91	0.056
		DCB similarity	0.11 ± 0.240	0.45	0.652
		DAB similarity	−0.03 ± 0.243	−0.13	0.900
Breeding success	53	**Intercept**	**−0.98 ± 0.225**	**−4.36**	**<0.001**
		**Year**	**−1.14 ± 0.463**	**−2.46**	**0.014**
		DBB similarity	0.61 ± 0.434	1.41	0.158
		DCB similarity	−0.13 ± 0.468	−0.29	0.773
		**DAB similarity**	**0.99 ± 0.452**	**2.19**	**0.029**
Offspring success	26	**Intercept**	**1.48 ± 0.551**	**2.69**	**0.007**
		Year	1.60 ± 0.906	1.76	0.078
		DBB similarity	−0.18 ± 0.826	−0.22	0.828
		DCB similarity	0.90 ± 0.874	1.03	0.303
		DAB similarity	0.27 ± 0.856	0.31	0.753
C. Genome-wide similarity					
Copulation success	89	**Intercept**	**−1.14 ± 0.132**	**−8.65**	**<0.001**
		**Year**	**−0.66 ± 0.275**	**−2.42**	**0.016**
		Familiarity	0.36 ± 0.216	1.67	0.094
		Male age	0.37 ± 0.235	1.59	0.111
		Similarity	0.19 ± 0.241	0.78	0.435
Breeding success	53	**Intercept**	**−1.10 ± 0.232**	**−4.73**	**<0.001**
		**Year**	**−1.78 ± 0.493**	**−3.62**	**<0.001**
		**Similarity**	**0.89 ± 0.448**	**1.99**	**0.046**
Offspring success	26	**Intercept**	**1.35 ± 0.561**	**2.41**	**0.016**
		Year	1.03 ± 0.965	1.07	0.284
		Similarity	0.72 ± 0.838	0.85	0.393

**Notes:**

Predictors in bold show coefficients that are statistically different from 0 at the 0.05 α level.

Predictor variables were standardized by subtracting the mean and dividing by two standard deviations (see Methods).

#### Advantage of particular alleles

Copulation success rates were higher in males that did not carry the DCB226 or DCB254 allele than males that did carry either of these alleles (Table 5). Conversely, males that carried the DCB266 allele were more likely to produce offspring than males without the allele (Table 5). Males that carried the DBB297 allele showed higher breeding success rates than males with other alleles, while males that carried the DAB289 allele showed reduced breeding success rates relative to males that did not carry the allele (Table 5). No particular alleles were found to influence offspring success (Table 5).

**Table 5:** Effect of carrying specific MHCII alleles on male copulation, breeding and offspring success.

Response variable	Locus	Allele*	*n* (0, 1, 2)	Slope ± SE	AIC_C_	δAIC_C_
Copulation success	DBB	297	18/3/0	−0.952 ± 0.491	96.9	–
		Base	–	–	98.7	1.82
		287	17/4/0	−0.192 ± 0.213	99.9	3.00
		289	10/11/0	0.047 ± 0.196	100.6	3.76
		277	5/11/5	−0.004 ± 0.194	100.7	3.82
	DCB	**266**	**18/3/0**	**−1.201 ± 0.492**	**94.6**	**–**
		**254**	**16/5/0**	**−0.560 ± 0.247**	**95.3**	**0.73**
		220	18/3/0	0.532 ± 0.323	98.0	3.36
		226	18/3/0	0.311 ± 0.213	98.6	3.96
		Base	–	–	98.7	4.07
		250	18/3/0	0.381 ± 0.282	98.9	4.27
		260	19/2/0	−0.343 ± 0.288	99.2	4.61
		256	9/9/3	−0.228 ± 0.195	99.3	4.70
		252	18/3/0	0.300 ± 0.260	99.4	4.76
		228	19/2/0	0.364 ± 0.361	99.7	5.05
	DAB	Base	–	–	98.7	–
		289	15/6/0	0.335 ± 0.237	98.7	0.01
		297	14/4/3	0.089 ± 0.198	100.5	1.80
		285	14/7/0	−0.038 ± 0.204	100.6	1.97
		287	18/3/0	−0.362 ± 0.310	99.3	0.62
		291	13/7/1	−0.213 ± 0.196	99.5	0.80
		293	17/4/0	−0.181 ± 0.219	100.0	1.31
Breeding success	DBB	**297**	**15/2/0**	**1.186 ± 0.427**	**67.0**	**–**
		Base	–	–	73.0	6.03
		289	7/10/0	−0.199 ± 0.325	74.7	7.65
		287	15/2/0	0.026 ± 0.327	75.0	8.02
		277	3/10/4	−0.024 ± 0.308	75.0	8.02
	DCB	**266**	**15/2/0**	**1.186 ± 0.427**	**67.0**	**–**
		260	16/1/0	1.180 ± 0.511	69.1	2.12
		254	13/4/0	0.883 ± 0.410	70.3	3.26
		Base	–	–	73.0	6.03
		228	15/2/0	0.099 ± 0.507	75.0	7.99
		220	14/3/0	0.017 ± 0.634	75.0	8.03
		226	15/2/0	−0.936 ± 0.396	69.0	2.02
		252	14/3/0	−0.902 ± 0.419	70.1	3.11
		250	15/2/0	−0.619 ± 0.452	73.1	6.07
		256	6/9/2	−0.031 ± 0.319	75.0	8.02
	DAB	**289**	**11/6/0**	**−0.999 ± 0.394**	**68.2**	**–**
		291	11/6/0	0.560 ± 0.315	71.9	3.62
		293	14/3/0	0.511 ± 0.344	72.8	4.59
		Base	–	–	73.0	4.80
		285	12/5/0	0.192 ± 0.30	74.6	6.39
		287	14/3/0	−0.282 ± 0.481	74.7	6.46
		297	11/3/3	0.010 ± 0.319	75.0	6.80
Offspring success	DBB	289	6/7/0	−0.863 ± 0.540	34.5	–
		297	11/2/0	−0.992 ± 0.617	34.6	0.03
		Base	–	–	35.2	0.67
		277	3/7/0	0.489 ± 0.576	36.4	1.92
		287	11/2/0	0.215 ± 0.512	37.0	2.49
	DCB	266	11/2/0	−0.992 ± 0.617	34.6	–
		Base	–	–	35.2	0.63
		228	11/2/0	0.825 ± 0.718	35.8	1.25
		250	11/2/0	0.824 ± 0.857	36.2	1.60
		226	11/2/0	0.744 ± 0.843	36.3	1.77
		256	4/8/0	−0.413 ± 0.512	36.5	1.97
		252	10/3/0	0.317 ± 0.689	37.0	2.42
		254	11/2/0	0.208 ± 0.563	37.1	2.50
		220	11/2/0	0.085 ± 1.361	37.2	2.63
		260	12/1/0	−0.011 ± 0.582	37.2	2.63
	DAB	Base	–	–	35.2	–
		285	8/5/0	−0.618 ± 0.488	35.6	0.36
		293	10/3/0	0.295 ± 0.531	36.9	1.69
		287	11/2/0	0.330 ± 0.691	37.0	1.77
		297	9/3/0	0.226 ± 0.510	37.0	1.80
		289	8/5/0	−0.084 ± 0.587	37.2	1.98
		291	8/5/0	−0.056 ± 0.476	37.2	1.99

**Notes:**

Only alleles that were present in more than one male were included.

Models shown in bold show strong evidence that the respective allele influences the corresponding response variable due to the AIC_C_ values ranking highly (≥2 AIC_C_) above the next best model and above the base* model.

*n* represents the number of males carrying 0, 1 or 2 copies of the specified allele.

* All models are generalized linear models with response variables fitted as binomial trials (see Methods). All allele models include base parameters such as age and year (see Methods) plus a 1/0 binary predictor for presence/absence of the specified allele. Base models only include base parameters.

## Discussion

This is the first study to examine both genome-wide and MHC-dependent mate choice preferences, in addition to non-genetic factors, at multiple stages of the mating process in captive koalas. We reconfirmed that both age and familiarity were determinants of mating success in this species (Bercovitch et al., 2006). There was evidence of genome-wide mate preferences as well as pre-copulatory and post-copulatory MHC-dependent mate choice under all three mate choice hypotheses, (A) quantity of MHC alleles; (B) genetic compatibility between mates; and (C) advantage of particular alleles (hypotheses reviewed in Kamiya et al., 2014; Setchell & Huchard, 2010). These results suggest that koalas use a combination of genetic, and non-genetic, mechanisms to select mates and optimize both the quantity and combination of alleles in their offspring.

### Non-genetic determinants of koala mate choice

Our analysis showed that koala copulation success is significantly influenced by male age and/or the age difference between males and females, in line with previous studies (Bercovitch et al., 2006). Studies in other species suggest that females may prefer to mate with older males, likely due to older males being of a higher genetic quality through viability selection (Manning, 1985; Trivers, 1972). In koalas, male size, bellowing and sternal scent secretions have been found to convey age-related information (Charlton et al., 2012; Salamon & Davies, 1998; Tobey et al., 2006), and so females may use visual, auditory and chemical cues to select mates based on age (Bercovitch et al., 2006). Here, we provide additional evidence that male age influences captive koala mate choice, although the precise chemical and auditory mechanisms by which females receive and utilize this information remain unclear (Ellis et al., 2015; Tobey, Nute & Bercovitch, 2009).

In addition to age, we also found that familiarity may promote copulation success in captive koalas. Other mammal mate choice studies show that females have a preference for more familiar males (Roberts & Gosling, 2004) and mating with familiar males leads to increased reproductive success (Martin & Shepherdson, 2012). This familiar male preference often arises in territorial scent-marking species, as females encounter scent marks of locally territorial males and select these males due to their ability to defend a territory (Rich & Hurst, 1998). Although koalas are a territorial scent-marking species (Allen et al., 2010), female koalas do not show a preference for locally territorial males in the wild (Ellis, Hale & Carrick, 2002). The familiarity trend in our study may be driven by pairing previously successful pairs together in subsequent years, although most (60%) of the pairings in our dataset were from first-time pairings. Further research, which directly examines the role of familiarity, is needed to confirm its influence in koala mate choice.

### Genetic determinants of koala mate choice

Previous research suggests that females of many species are often more attracted to heterozygous males, and heterozygosity has been linked to numerous advantages such as greater sexual ornamentation, mating success and overall reproductive success (reviewed in Kempenaers, 2007). Despite these advantages, genome-wide heterozygosity did not influence mating success in our captive koalas. Some species also display a preference for dissimilar individuals, which may reduce inbreeding and increase genetic diversity of offspring (Ferrandiz-Rovira et al., 2016; Kempenaers, 2007). In contrast, we found a positive association between genome-wide similarity and breeding success, suggesting female koalas are more likely to produce offspring with males that are more genetically similar overall. A recent review showed mating with similar individuals can allow populations to adapt more quickly to virulent diseases and parasites (Campbell et al., 2017). Assortative mate preferences may help protect koala populations from threatening infectious diseases such as chlamydia (Polkinghorne, Hanger & Timms, 2013) and koala retrovirus (Denner & Young, 2013), and should be examined further. Although similar numbers of neutral microsatellite markers have been used in recent studies to examine genome-wide mating preferences (Ferrandiz-Rovira et al., 2016; Huchard et al., 2013), estimates of genome-wide diversity based on 15 microsatellites may not be sufficient, and larger numbers of markers should be employed in future studies to provide more accurate measures of genome-wide diversity (Miller et al., 2014).

In addition to genome-wide mating preferences, many species select mates based on MHC (Table 1). Consistent with these studies, we found that koala mating success showed a significant association with male heterozygosity and pair similarity at MHC loci, as well as the presence or absence of particular MHC alleles. In contrast to the quantity of alleles hypothesis, males that were less heterozygous at MHC-linked loci showed a greater rate of copulation success. This indicates that female koalas prefer to copulate with males that have fewer alleles at MHC loci, particularly at DAB loci (we note that individual locus heterozygosity and overall MHC heterozygosity are related and do not provide multiple lines of evidence). Interestingly, among those males that did copulate, breeding success was higher for more heterozygous males. This implies that females are more likely to produce offspring when breeding with males of higher heterozygosity, than when breeding with males of lower heterozygosity. The standardized slopes of the trends at each stage were of similar magnitude (Table 3), suggesting that the effect of heterozygosity is similar at both mate choice stages. Assessed together, these results reflect differences in the pre-copulatory and post-copulatory MHC-dependent choice mechanisms in koalas.

Vertebrate females are known to select sperm based on heterozygosity or diversity at MHC loci (Wedekind, 2002; Winternitz et al., 2013). Males heterozygous at MHC loci show significantly greater fertilization success relative to homozygous males (Skarstein et al., 2005). In koalas, males with low heterozygosity at MHC loci overall (particularly at DAB loci) have a higher probability of copulating; however, more heterozygous males experience a fertilization advantage, so that their copulations are more likely to result in the production of offspring. While the benefits of breeding with heterozygous males can be explained by the increased antigenic peptide repertoire and immunocompetence of heterozygotes (Kamiya et al., 2014; Landry et al., 2001), we are unaware of any other reports where less-heterozygous males have a higher copulation success rate. Future work should investigate whether this unexpected relationship may be driven by an unmeasured male trait that is correlated with MHC heterozygosity.

Contrary to many previous findings under the genetic compatibility hypothesis (Table 1), captive koala pairs that were more similar at the MHCII DAB-linked locus had greater breeding success than less similar pairs. Female koalas were more likely to produce offspring with males that shared alleles at DAB loci, which is consistent with a greater reliance on post-copulatory MHC-dependent mechanisms of mate choice. Numerous studies have shown that females select sperm based on the genetic dissimilarity of mates (Olsson et al., 1996; Thuman & Griffith, 2005), particularly at MHC loci (Schwensow, Eberle & Sommer, 2008; Yeates et al., 2009). A preference for mates that are more similar at MHC loci may evolve in response to disadvantages associated with mating with individuals that are too dissimilar, including increased risk of autoimmune disorders due to suboptimal T-cell selection (Kaufman, Völk & Wallny, 1995), reduced recognition of foreign peptides due to T-cell loss (Vidovi & Matzinger, 1988), and disruption of co-adapted gene complexes (Hendry et al., 2000).

Studies have shown that, in some circumstances, carrying multiple copies of the same MHC allele allows for higher disease resistance (Grimholt et al., 2003; Nuismer, Otto & Blanquart, 2008). However, MHC assortative mating may make populations more vulnerable to future disease outbreaks or other stochastic events (Campbell et al., 2017). We suggest that female koalas might not solely choose more-similar mates but may rather optimize the quantity and combination of MHC alleles in the offspring (see also Milinski, 2006). A similarly complex mate choice mechanism has been demonstrated in sticklebacks (*Gasterosteus aculeatus*), whereby females prefer to mate with males with genotypes that, when combined with their own MHC alleles, will produce offspring with an optimal number of alleles and provide the highest possible resistance against common parasites (Milinski, 2003). A study of the numbers and combinations of MHC alleles in offspring with reference to the parents’ MHC is needed to confirm whether this mechanism exists in koalas.

In line with the advantage of particular alleles hypothesis, we found that the presence of certain MHC-linked microsatellite alleles was associated with increased or decreased mating success, whilst other alleles showed no association. Since the MHC-linked microsatellites are found in non-coding regions of the genome (and likely have no direct functional consequences), this finding suggests that females are selecting for and/or against males that carry the respective MHC alleles. Although some of these models appear to provide strong evidence for the influence of certain alleles on mating success, we note cautious interpretation of these findings is warranted as small sample sizes and subject: predictor ratios limit the reliability of the models (Table 5). Even so, by using a multimodel inference framework, i.e., ranking models under information theory, we provide support for the hypothesis that some MHC-linked microsatellite alleles are associated with mating success. This is probably because they are in linkage disequilibrium with important functional MHC sequence variants that potentially play a role in disease resistance and susceptibility to common infectious diseases such as Chlamydia (Lau, Canfield & Higgins, 2012).

Mate choice influences offspring viability in a variety of species, particularly when mating preferences are MHC-dependent (Agbali et al., 2010; Von Schantz et al., 1996). In koalas there was no association between offspring survival and mate choice preferences, however, data on offspring survival was only present for koala pairings that produced offspring (*n* = 13 males, 26 pairs, 28 offspring in total). Investigation into early joey loss will ascertain if observed mating preferences produce offspring with optimal MHC, as successful matings resulting in offspring with optimal immunity will increase offspring viability whilst suboptimal MHC will, in theory, produce early offspring deaths.

By using MHC-linked microsatellites we were able to examine three families of MHC loci to look for general patterns as well as locus-specific patterns. The majority of MHC-dependent mate choice studies in the current literature often only examine a single locus due to the limitations of MHC typing techniques (Kamiya et al., 2014). Our data indicate that some loci may play a larger role in mate choice than others, and different loci may act in different ways, further indicating the importance of examining multiple MHC loci. Huchard et al. (2013) found that female grey mouse lemurs (*Microcebus murinus*) chose males based on a particular MHCII locus that was under stronger diversifying selection than other MHCII loci. Similarly, DAB loci in the koala have previously been found to be under stronger selection than other MHCII loci (Lau et al., 2013), which may explain the strong effect of DAB on koala mate choice in the current study. Multi-locus approaches are vital in gaining a holistic understanding of MHC-dependent mate choice mechanisms (Kamiya et al., 2014) and can be easily achieved using MHC-linked microsatellite markers. Although numerous studies have confirmed MHC-linked microsatellite markers as a good proxy for MHC diversity in other species (Cheng & Belov, 2012; Cheng et al., 2009a; Crouau-Roy et al., 1996), this association needs to be confirmed in the koala.

## Conclusions

In conclusion, pair incompatibility is an important contributing factor for why many captive breeding programs are failing to reach program goals (Lees & Wilcken, 2009). We found a significant decrease in the copulation and breeding success of San Diego Zoo koalas, indicating a potential risk to future sustainability. The age of males and familiarity of pairs played a role in mate choice. We also found evidence that genome-wide similarity and MHC-diversity were associated with mating success, and mate choice mechanisms may consequently be contributing to reduced copulation rates and breeding success. The importance of examining both genetic and non-genetic determinants of mate choice in captive populations is highlighted and will help aid future pairing recommendations in captive facilities. This has important implications, not only for the management of captive koalas, but for all conservation breeding initiatives for threatened species.

## Supplemental Information

10.7717/peerj.5438/supp-1 Supplemental Information 1 Supplementary information. Supplementary methods, tables and figures.

10.7717/peerj.5438/supp-2 Supplemental Information 2 Supplemental data. Raw data files with a key and information on how to perform each analysis.

10.7717/peerj.5438/supp-3 Supplemental Information 3 Supplemental R Code 1. Script for analysing the San Diego Zoo pairing data and non-genetic factors of mate choice.

10.7717/peerj.5438/supp-4 Supplemental Information 4 Supplemental R Code 2. Script for calculating standardised heterozygosities, and determining whether MHC and non-MHC markers were correlated.

10.7717/peerj.5438/supp-5 Supplemental Information 5 Supplemental R Code 3. Script for testing the quantity of alleles mate choice hypothesis.

10.7717/peerj.5438/supp-6 Supplemental Information 6 Supplemental R Code 4. Script for testing the genetic compatibility mate choice hypothesis.

10.7717/peerj.5438/supp-7 Supplemental Information 7 Supplemental R Code 5. Script for testing the advantage of particular alleles mate choice hypothesis.

## Additional Information and Declarations

### Data Availability

The following information was supplied regarding data availability: The raw data are provided in a Supplemental Files.
