# Supplementary material for: Disentangling the mechanisms of mate choice in a captive koala population
Source: PeerJ. 2018 Aug 21;6:e5438. doi: 10.7717/peerj.5438 (PMC6108315; doi:10.7717/peerj.5438)
Supplement: Supplemental Information 1 — Supplementary methods, tables and figures. [file peerj-06-5438-s001.docx]

**SUPPLEMENTAL INFORMATION**

Brandies PA, Grueber CE, Ivy JA, Hogg CJ and Belov K. “Disentangling the mechanisms of mate choice in a captive koala population”.

*Initial primer screening and optimisation methods*

Twelve DNA samples from the Australian Lone Pine Sanctuary koala breeding population were also available from a previous study, and were found to be polymorphic at MHC loci by cloning and sequencing (Cheng et al. 2017). Screening and optimisation of microsatellite primers was performed on these samples, because the quantity of DNA from the San Diego Zoo samples was limited and the extent of polymorphism in the study population was unknown. Though this approach may have introduced a level of ascertainment bias to the study, we predicted that the effects would be minimal as the Lone Pine koala population is likely to be more polymorphic than the closed San Diego Zoo population, due to regular supplementation of wild rehabilitation koalas into the Lone Pine population.

*Marker diversity*

MICROCHECKER (Van Oosterhout et al. 2004) was used to screen the genotyping data for evidence of null alleles. Number of alleles, observed (H_o_) heterozygosity and expected (H_e_) heterozygosity were calculated for each locus using GenAlEx (Peakall & Smouse 2012; Peakall & Smouse 2006). Hardy-Weinberg tests were performed with Genepop (Raymond & Rousset 1995b; Rousset 2008) using the Markov chain method (Guo & Thompson 1992). Genotypic linkage disequilibrium was determined through log likelihood ratio statistics (G-tests) in Genepop using a Markov chain algorithm (Raymond & Rousset 1995a), with multiple tests corrected for using Holm’s sequential Bonferroni adjustment (Abdi 2010). We also performed all of the above analyses again on a subset of the population that excluded any sibling (full or half), or parent-offspring relationships, since the presence of many closely related individuals in the population may explain apparent patterns of linkage disequilibrium or deviations from Hardy-Weinberg equilibrium (Excoffier & Slatkin 1998; Robertson & Hill 1984).

MHC-linked PhciDBB001M3 showed significant deviations form Hardy-Weinberg equilibrium (Table S6) and showed strong evidence of linkage disequilibrium with PhciDCBM1 (p < 0.001). Upon retesting a subset of unrelated individuals, all three MHC-linked markers were in Hardy-Weinberg equilibrium and linkage equilibrium. Non-MHC markers Pcin23 and Pcv26 showed significant deviation from Hardy-Weinberg equilibrium in the complete study population but were found to be in Hardy-Weinberg equilibrium in the subset of unrelated individuals (Table S6). All non-MHC markers were in linkage equilibrium following sequential Bonferroni correction.

**Table S1.** Comparison of complete koala data and sampled koala data (1984-2012)

|  | Complete Koala Data | Sampled Koala Data^†^ |
| --- | --- | --- |
| Total number of pairing events | 964 | 471 |
| Number of unique male-female pair combinations | 225 | 89 |
| Number of breeding recommendations^*^ | 400 | 173 |
| Number of males paired | 46 | 21 |
| Number of females paired | 49 | 27 |

**^*^** Number of breeding recommendations = sum of the number of unique male-female pair combinations per year over the 28 years

^†^ Subset of the complete dataset including only the study population of koalas which have DNA samples provided

**Table S2**. Correlation matrix of predictor variables

|  | Year | Female Age | Male Age | Age Difference | Familiarity |
| --- | --- | --- | --- | --- | --- |
| Year |  |  |  |  |  |
| Female Age | 0.02  p = 0.453 |  |  |  |  |
| Male Age | 0.40  p < 0.001 | 0.03  p = 0.368 |  |  |  |
| Age Difference | 0.30  p < 0.001 | **-0.58**  **p < 0.001** | **0.76**  **p < 0.001** |  |  |
| Familiarity | 0.08  p = 0.01 | 0.43  p < 0.001 | 0.26  p < 0.001 | -0.08  p = 0.013 |  |

Correlation method = Spearman’s rank correlation

Moderate to strong correlations are bolded

**Table S3.** PCR thermocycling conditions

| **Markers** | PhciDBB001M3, PhciDCBM1, MHCIIDAB001M1, Pcin05, Pcin08, Pcin11, Pcin20, Pcin21, Pcin22, Pcin23 | | | Pcv31, Pcv25.2, Pcv30, Pcv25.1 | | | Pcv24.2, Pcv26, Phc13, Phc11 | | |
| --- | --- | --- | --- | --- | --- | --- | --- | --- | --- |
| **Step** | **Temp** | **Time** | **Cycles** | **Temp** | **Time** | **Cycles** | **Temp** | **Time** | **Cycles** |
| Initial Denaturation | 95˚C | 5 min | 1 | 95˚C | 5 min | 1 | 95˚C | 5 min | 1 |
| Denaturing | 95˚C | 30 s | 25 | 94˚C | 30 s | 30 | 94˚C | 30 s | 20 |
| Annealing | 60˚C | 90 s |  | 55˚C | 45 s |  | 70˚C | 45 s |  |
| Elongation | 72˚C | 30 s |  | 72˚C | 30 s |  | 72˚C | 45 s |  |
| Denaturation | - | - | - | 94˚C | 30 s | 8 | 94˚C | 30 s | 15 |
| Annealing | - | - |  | 53˚C | 45 s |  | AT* | 45 s |  |
| Elongation | - | - |  | 72˚C | 45 s |  | 72˚C | 45 s |  |
| Final Extension | 60˚C | 30 min | 1 | 72˚C | 30 min | 1 | 72˚C | 10 min | 1 |

*Annealing temperature was 55˚C for Pcv24.2 and Pcv26, and 50˚C for Phc11 and Phc13.

**Table S4.** Non-MHC microsatellite markers with corresponding information

| **Marker Name** | **Tag** | **Multiplex** | **Reference** |
| --- | --- | --- | --- |
| Pcin05 | CAG-tag | M3 | Dennison et al. (2017) |
|  |  |  |  |
| Pcin08 | CAG-tag | M1 | Dennison et al. (2017) |
|  |  |  |  |
| Pcin11 | CAG-tag | M2 | Dennison et al. (2017) |
|  |  |  |  |
| Pcin20 | CAG-tag | M1 | Dennison et al. (2017) |
|  |  |  |  |
| Pcin21 | CAG-tag | M2 | Dennison et al. (2017) |
|  |  |  |  |
| Pcin22 | CAG-tag | M2 | Dennison et al. (2017) |
|  |  |  |  |
| Pcin23 | CAG-tag | M3 | Dennison et al. (2017) |
|  |  |  |  |
| Pcv24.2 | None | - | Cristescu et al. (2009) |
|  |  |  |  |
| Pcv25.1 | M13 tail | - | Cristescu et al. (2009) |
|  |  |  |  |
| Pcv25.2 | M13 tail | - | Cristescu et al. (2009) |
|  |  |  |  |
| Pcv26 | None | - | Cristescu et al. (2009) |
|  |  |  |  |
| Pcv30 | M13 tail | - | Cristescu et al. (2009) |
|  |  |  |  |
| Pcv31 | M13 tail | - | Cristescu et al. (2009) |
|  |  |  |  |
| Phc11 | None | - | Houlden et al. (1996) |
|  |  |  |  |
| Phc13 | None | - | Houlden et al. (1996) |
|  |  |  |  |

**Table S5.** MHC and non-MHC marker diversity for the complete study population and a subset of the study population which excludes any sibling (full or half) or parent-offspring relationships

|  | **Marker Name** | **Est. Freq.** | **Study population** | | | | | | **Unrelated Subset** | | | | | |
| --- | --- | --- | --- | --- | --- | --- | --- | --- | --- | --- | --- | --- | --- | --- |
|  |  |  | **Na** | **Product Length** | **n** | **H_o_** | **H_e_** | **P** | **Na** | **Product Length** | **n** | **H_o_** | **H_e_** | **P** |
| MHC | PhciDBB001M3 | 0.011 | 7 | 277-303 | 70 | 0.571 | 0.589 | <0.01 | 5 | 277-297 | 24 | 0.542 | 0.537 | 0.149 |
|  | PhciDCBM1 | 0.000 | 13 | 220-266 | 70 | 0.871 | 0.848 | 0.130 | 11 | 220-266 | 24 | 0.833 | 0.862 | 0.919 |
|  | MHCIIDAB001M1 | 0.000 | 9 | 277-297 | 70 | 0.871 | 0.829 | 0.622 | 9 | 277-297 | 24 | 0.833 | 0.832 | 0.412 |
| Non-MHC | Pcin05 | 0.005 | 7 | 185-209 | 70 | 0.757 | 0.763 | 0.711 | 7 | 185-209 | 24 | 0.667 | 0.693 | 0.110 |
|  | Pcin08 | 0.057 | 8 | 144-174 | 70 | 0.557 | 0.627 | 0.089 | 7 | 144-170 | 24 | 0.500 | 0.682 | 0.069 |
|  | Pcin11 | 0.018 | 7 | 147-163 | 70 | 0.729 | 0.754 | 0.491 | 6 | 147-163 | 24 | 0.750 | 0.770 | 0.841 |
|  | Pcin20 | -0.090 | 2 | 264-267 | 70 | 0.171 | 0.157 | 1.000 | 2 | 264-267 | 24 | 0.208 | 0.187 | 1.000 |
|  | Pcin21 | 0.011 | 6 | 229-250 | 70 | 0.743 | 0.753 | 0.757 | 6 | 229-250 | 24 | 0.708 | 0.748 | 0.395 |
|  | Pcin22 | -0.056 | 7 | 316-337 | 70 | 0.857 | 0.780 | 0.349 | 7  p = 0.073 | 316-337 | 24  p = 0.073 | 0.917 | 0.811 | 0.235 |
|  | Pcin23 | 0.069 | 5 | 95-107 | 70 | 0.500 | 0.558 | 0.049 | 7 | 95-107 | 24 | 0.667 | 0.693 | 0.735 |
|  | Pcv24.2 | -0.059 | 8 | 196-212 | 51 | 0.882 | 0.792 | 0.205 | 7 | 198-212 | 17 | 0.882 | 0.761 | 0.296 |
|  | Pcv25.1 | -0.052 | 4 | 83-101 | 54 | 0.722 | 0.658 | 0.936 | 3 | 83-101 | 17 | 0.882 | 0.642 | 0.286 |
|  | Pcv25.2 | 0.033 | 4 | 179-187 | 53 | 0.377 | 0.399 | 0.313 | 4 | 179-187 | 17 | 0.353 | 0.424 | 0.270 |
|  | Pcv26 | -0.072 | 7 | 196-212 | 51 | 0.804 | 0.724 | 0.009 | 6 | 196-212 | 16 | 0.750 | 0.785 | 0.162 |
|  | Pcv30 | -0.022 | 5 | 192-216 | 53 | 0.717 | 0.679 | 0.803 | 4 | 192-208 | 17 | 0.706 | 0.663 | 0.900 |
|  | Pcv31 | -0.035 | 9 | 220-252 | 54 | 0.759 | 0.718 | 0.589 | 9 | 220-252 | 17 | 0.941 | 0.789 | 0.221 |
|  | Phc11 | 0.028 | 9 | 210-236 | 53 | 0.698 | 0.743 | 0.686 | 7 | 212-236 | 17 | 0.706 | 0.725 | 0.153 |
|  | Phc13 | 0.003 | 10 | 97-125 | 52 | 0.827 | 0.832 | 0.251 | 10 | 97-125 | 17 | 0.647 | 0.808 | 0.025 |

Est. Freq. = Oosterhout measure of estimated null allele frequencies, Na = number of observed alleles, n = number of individuals genotyped, H_o_ = observed heterozygosity, H_e_ = expected heterozygosity, P = tests of deviation from Hardy-Weinberg equilibrium.

**Figure S1. Comparison of average copulation, breeding and offspring success rates (± SE) per year (n = 29) for the San Diego Zoo complete koala data (light grey) and sampled koala data (dark grey).** GLMs with binomial distribution revealed no significant differences in average copulation, breeding and offspring success rates across the complete dataset and sampled dataset (copulation success: z = -0.724, p = 0.469; breeding success: z = -1.187, p = 0.235; offspring success: z = 0.573, p = 0.567).

**Figure S2. Frequency histograms showing the number of years each pair was represented in the datasets containing A) all pairings (mean = 1.8, SD = 1.30), B) pairings that resulted in successful copulations (mean = 1, SD = 1.04) and C) pairings that resulted in offspring (mean = 0.6, SD = 0.87)**. It was found that 60% of all pairs, 67% of the pairs resulting in copulation, and 72% of the pairs resulting in offspring were only represented in 1 year of the respective datasets.

**Literature cited in supplemental material**

Abdi H. 2010. Holm’s sequential Bonferroni procedure. *Encyclopedia of Research Design* 1.

Cheng Y, Polkinghorne A, Gillett A, Jones EA, O’Meally D, Timms P, and Belov K. 2017. Characterisation of MHC class I genes in the koala. *Immunogenetics*. 10.1007/s00251-017-1018-2

Cristescu R, Cahill V, Sherwin WB, Handasyde K, Carlyon K, Whisson D, Herbert CA, Carlsson BLJ, Wilton AN, and Cooper DW. 2009. Inbreeding and testicular abnormalities in a bottlenecked population of koalas (*Phascolarctos cinereus*). *Wildlife Research* 36:299-308.

Dennison S, Frankham G, Neaves L, Flanagan C, FitzGibbon S, Eldridge M, and Johnson R. 2017. Population genetics of the koala (*Phascolarctos cinereus*) in north-eastern New South Wales and south-eastern Queensland. *Australian Journal of Zoology* 64:402-412.

Excoffier L, and Slatkin M. 1998. Incorporating genotypes of relatives into a test of linkage disequilibrium. *The American Journal of Human Genetics* 62:171-180. 10.1086/301674

Guo SW, and Thompson EA. 1992. Performing the exact test of Hardy-Weinberg proportion for multiple alleles. *Biometrics* 48:361-372. 10.2307/2532296

Houlden BA, England P, and Sherwin WB. 1996. Paternity exclusion in koalas using hypervariable microsatellites. *The Journal of Heredity* 87:149-152. 10.1093/oxfordjournals.jhered.a022972

Peakall R, and Smouse PE. 2012. GenALEx 6.5: Genetic analysis in Excel. Population genetic software for teaching and research-an update. *Bioinformatics* 28:2537-2539. 10.1093/bioinformatics/bts460

Peakall ROD, and Smouse PE. 2006. GenALEx 6: Genetic analysis in Excel. Population genetic software for teaching and research. *Molecular Ecology Notes* 6:288-295. 10.1111/j.1471-8286.2005.01155.x

Raymond M, and Rousset F. 1995a. An exact test for population differentiation. *Evolution* 49:1280-1283.

Raymond M, and Rousset F. 1995b. GENEPOP (Version-1.2) - Population-genetics software for exact tests and ecumenicism. *Journal of Heredity* 86:248-249. 10.1093/oxfordjournals.jhered.a111573

Robertson A, and Hill WG. 1984. Deviations from Hardy-Weinberg proportions: Sampling variances and use in estimation of inbreeding coefficients. *Genetics* 107:703-718.

Rousset F. 2008. GENEPOP'007: A complete re-implementation of the GENEPOP software for Windows and Linux. *Molecular Ecology Resources* 8:103-106. 10.1111/j.1471-8286.2007.01931.x

Van Oosterhout C, Hutchinson WF, Wills DP, and Shipley P. 2004. MICRO‐CHECKER: software for identifying and correcting genotyping errors in microsatellite data. *Molecular Ecology Notes* 4:535-538.
